# Effectiveness and Safety of Lumbar Erector Spinae Plane Block Versus No Locoregional Block in Hip Surgery: Protocol for a Randomized Controlled Trial

**DOI:** 10.2196/75854

**Published:** 2025-09-15

**Authors:** Laura García-Reza, Sergio Raposeiras, Javier P Loureiro, Rafael Pita-Romero, Juan Jose Amate Pena, Miguel Angel Pereira Loureiro

**Affiliations:** 1 Division of Anaesthetics, Pain Medicine and Intensive Care University Hospital Álvaro Cunqueiro Vigo Spain; 2 TALIONIS Research group Information and Communications Technology Research Center A Coruña University A Coruña Spain

**Keywords:** hip fracture, hip osteoarthritis, lumbar erector spinae plane block, pain, morphine

## Abstract

**Background:**

Ultrasound-guided regional anesthesia in hip surgery has been shown to reduce the need for opioids and conventional analgesics, facilitate ambulation and early recovery, improve respiratory dynamics, and decrease the incidence of venous thrombosis and pneumonia. Lumbar erector spinae plane block (L-ESPB) has been proposed as a novel ultrasound-guided locoregional technique to relieve pain in hip surgery; however, the supporting evidence remains scarce.

**Objective:**

This study aims to provide evidence on the role of L-ESPB in perioperative pain management during hip surgery. We hypothesize that patients receiving L-ESPB compared to those without block will have lower opioid consumption and less pain after hip surgery. The primary outcome is the difference in pain intensity, measured using the visual analog scale, at 2 hours postoperatively between the L-ESPB group and the control group. We will compare postoperative analgesic needs and opioid consumption in both groups, assess the technical ease of performing L-ESPB, and record any side effects in both treatment arms. Finally, we will evaluate the level of patient satisfaction.

**Methods:**

This is a pragmatic, single-center, parallel-group randomized controlled trial. After patients provide informed consent, they will be randomly assigned in a 1:1 ratio to receive either ultrasound-guided L-ESPB or conventional intravenous analgesia. A total of 180 patients (n=90, 50% in each group) will be enrolled. Data analysis will be performed using SPSS software.

**Results:**

This clinical trial was approved by the European Medicines Agency on May 27, 2024, following approval by the local ethics committee earlier that month. Patient recruitment took place between June 2024 and May 2025. Statistical analysis is currently ongoing, and final results are expected to be submitted for publication in early 2026.

**Conclusions:**

This clinical trial will enable us to assess the analgesic effectiveness of L-ESPB in hip surgery. We will also evaluate its safety and potential adverse effects compared with conventional analgesia and opioid consumption.

**Trial Registration:**

ClinicalTrials.gov NCT06567522; https://www.clinicaltrials.gov/study/NCT06567522

**International Registered Report Identifier (IRRID):**

DERR1-10.2196/75854

## Introduction

### Background

Hip surgery accounts for a high percentage of both emergency and elective surgical procedures in hospitals. Regardless of surgery being prescribed to treat a fracture or coxarthrosis, patients are usually older adults with multiple associated comorbidities [1]. In this patient population, there is a tendency to undertreat pain for fear of the side effects and pharmacological interactions of conventional analgesic drugs [2].

Ultrasound-guided regional anesthesia in orthopedic and trauma surgery has been shown to reduce the need for opioids and conventional analgesics, facilitate ambulation and early recovery, improve respiratory dynamics, and decrease the incidence of venous thrombosis and pneumonia [3,4].

Numerous ultrasound-guided peripheral nerve blocks have been evaluated in hip surgery, although no single analgesic technique has yet been established as the gold standard. Examples include the femoral nerve block [5], fascia iliaca block [6], and the more recently introduced pericapsular nerve group (PENG) block [7]. Although these blocks are considered safe, they are performed close to nerves and important vessels, such as the femoral artery, and are therefore not without risk. Furthermore, they do not provide complete analgesia of the hip because they do not cover, for example, the obturator nerve [8], or, in the case of the PENG block, cutaneous branches [9].

The lumbar erector spinae plane block (L-ESPB) remains a relatively novel technique, particularly in hip surgery. Several publications have reported its utility in this context; however, all are limited to case series and lack standardized clinical trials. Tulgar and Senturk [10] and Tulgar et al [11] have described extensive and long-lasting pain relief after L-ESPB in hip arthroplasty. Conversely, Chan et al [12] found that L-ESPB did not reduce pain or opioid consumption in patients undergoing hip surgery.

### Objectives

Given the lack of high-quality clinical trials evaluating L-ESPB in hip surgery, this study is warranted. Our aim is to provide evidence that may help define the role of this technique in perioperative pain management, focusing on patients more vulnerable to opioid-related adverse effects.

We hypothesize that patients receiving L-ESPB, compared to those who do not, will experience lower opioid consumption and reduced postoperative pain after hip surgery. If L-ESPB demonstrates these benefits, it could represent a valuable addition to multimodal analgesia strategies, particularly for older adults or patients who are frail and thus more vulnerable to opioid-related side effects. Furthermore, the technical simplicity and safety profile of L-ESPB make it an attractive option for widespread clinical use, including in resource-limited settings.

## Methods

### Trial Design

This is a pragmatic, single-center, parallel-group, low-intervention randomized controlled trial comparing the analgesic efficacy of L-ESPB versus no block in the postoperative period of hip surgery. This protocol follows the SPIRIT (Standard Protocol Items: Recommendations for Interventional Trials) guidelines. This trial has been registered on ClinicalTrials.gov (NCT06567522).

### Study Setting

The study is conducted in a hospital setting, and we are recruiting patients scheduled for hip surgery due to either osteoarthritis or fracture.

### Eligibility Criteria

#### Inclusion Criteria

This study will include adult patients (aged >18 y) of both sexes scheduled for hip surgery, classified as American Society of Anesthesiologists (ASA) I to III according to the ASA (where ASA I indicates low anesthetic risk and ASA IV indicates high anesthetic risk). Eligible patents must provide written informed consent and be able to understand and use the visual analog scale (VAS) for pain assessment.

#### Exclusion Criteria

Patients will be excluded if they have contraindications to the technique or drugs used, if the block cannot be technically performed, if they have severe cognitive impairment or documented mental disabilities, or if they are already enrolled in another clinical trial.

### Randomization

Patients who consented to participate were randomly assigned to two groups: (1) experimental (L-ESPB will be performed at the L3-L4 level using 30 mL of 0.25% levobupivacaine) and (2) control. Randomization was performed using a computer-generated 1:1 balanced sequence, with both the sequence and group assignments concealed. This method is currently recommended as the standard for clinical trials.

The use of the locoregional technique makes blinding impossible for both patients and anesthetists. However, outcome assessment will be blinded: nurses collecting data in the immediate postoperative period and during subsequent ward care will be unaware of group assignments. To minimize blinding bias, we will use rigorous and uniform protocols to measure outcomes regardless of group assignment. In addition, a statistical analysis will be conducted with masked group labels (group A and group B) so that the analyst will remain unaware of treatment allocation. Blinded outcome assessment and independent statistical analysis are intended to minimize observational bias.

### Intervention

This is a clinical trial designed and conducted by researchers without industry involvement. It follows a pragmatic, single-center, parallel-group, low-intervention, nonblinded randomized controlled design, with blinded assessment of the objectives. All patients scheduled to undergo emergency or elective hip surgery who meet the inclusion criteria and none of the exclusion criteria will be included. The main variable will be pain control (assessed using the VAS). There will be 2 groups of patients (one receiving ultrasound-guided L-ESPB at the L3-L4 level with 30 mL of 0.25% levobupivacaine and a control group receiving conventional intravenous analgesia).

L-ESPB will be performed using a portable ultrasound machine with a linear or convex transducer and a 22G Pajunk needle (Pajunk GmbH Medizintechnologie; 50 mm or 80 mm, depending on block depth). The patient will be placed in the lateral decubitus position, with the side to be blocked facing up. We will locate the transverse processes at the L3-L4 level with a parasagittal approach. The 22G needle (50 mm or 80 mm, depending on the target depth) will then be inserted from cranial to caudal in plane with the ultrasound probe. We will advance through the erector spinae muscles, subsequently performing hydrodissection, followed by injection of 30 mL of 0.25% levobupivacaine after negative aspiration is confirmed.

**Figure 1:**
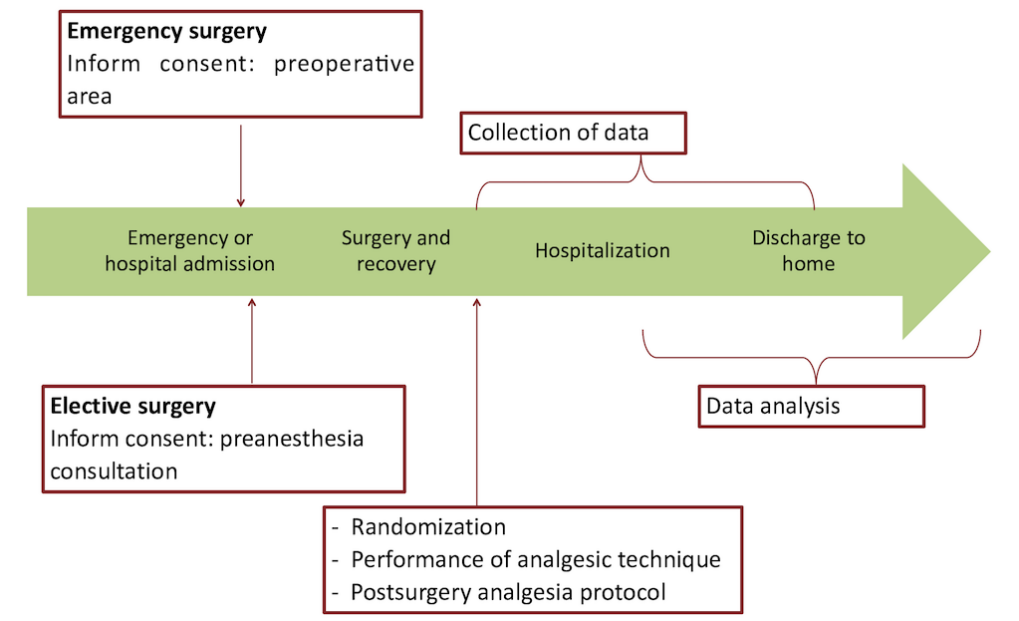
Study timeline. OR: operating room; PACU: postanesthesia care unit.

For patients receiving the locoregional technique, zero hour is defined as the earliest time within the first 2 postoperative hours when a VAS score greater than 1 is reported (Figure 1).

**Figure 2:**
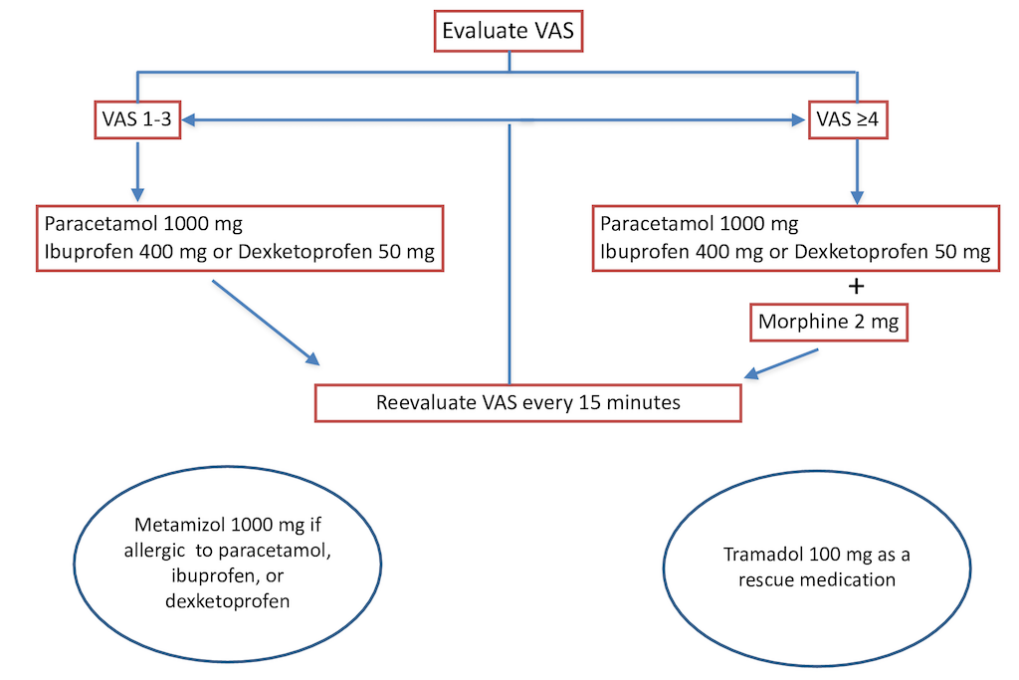
Analgesic protocol. VAS: visual analog scale.

An analgesic postoperative protocol will be applied. For those receiving the locoregional technique, 1000 mg of paracetamol will be administered every 8 hours, along with either 50 mg of dexketoprofen or 400 mg of ibuprofen every 8 hours. For patients allergic to nonsteroidal anti-inflammatory drugs or paracetamol, or when rescue medication is needed, 1000 mg of metamizol every 8 hours will be prescribed. Tramadol is also available as rescue medication. If the VAS score is equal to or greater than 4, we will concurrently prescribe 2 mg of morphine, followed by reassessment after 15 minutes. If the VAS score remains equal to or greater than 4 after delivering the bolus, the same morphine dose will be repeated, with subsequent reassessment every 15 minutes, up to a maximum of 12 mg of morphine in 24 hours (Figure 2). This analgesic protocol will also be applied to patients in the control group.

### Variables Studied

The main variable is the VAS score, recorded at 30 minutes and at 2, 6, 12, 24, and 48 hours postoperatively. We will also assess adverse effects related to the locoregional block and opioid use, the level of patient satisfaction, and the technical ease of performing the block.

Sociodemographic variables will include age, sex, height, weight, ASA classification, date of admission, date of surgery, type of surgery (prosthesis or osteosynthesis), type of anesthesia, and preblock VAS score.

The technical ease of performing the block will be classified as “good” when we are able to clearly distinguish the anatomical structures necessary for locoregional analgesia; “fair” when the structures are intuited but not clearly defined; and “poor” when we are unable to distinguish any anatomical structure that would allow us to perform the block, which constitutes an exclusion criterion for participation in the study. Forty-eight hours after the procedure, the level of patient satisfaction with the quality of analgesic treatment will be recorded as “satisfied” if the patient considers pain management adequate or “dissatisfied” if pain management is considered inadequate.

### Outcomes

#### Primary Outcome

The primary outcome is the difference in pain intensity between the L-ESPB group and the control group, measured using the VAS at 2 hours postoperatively.

#### Secondary Outcome

We will compare postoperative analgesic requirements and opioid consumption between the L-ESPB group and the control group. In the L-ESP block group, the technical ease of performing the block will be assessed and classified as good, fair, or poor. We will also compare the occurrence of side effects and the level of patient satisfaction between both groups.

### Sample Size

Considering a reduction of at least 1 point on the VAS, with an expected SD of 2, a statistical power of 90%, and a type I error of 5%, we calculated a minimum sample size of 86 patients per group (total N=172). To account for potential losses, we increased the sample size by 5%. At the time this protocol was developed, no clinical trials had evaluated this type of block in hip surgery. Therefore, we chose a 1-point decrease on the VAS, aiming to be both conservative and pragmatic, which resulted in a larger sample size compared to similar studies of other locoregional blocks in hip surgery [8,13].

Recruitment is planned over 18 months.

### Statistical Analysis

The analysis will be performed using SPSS software (IBM Corp). Descriptive statistics will be presented for all collected variables: frequencies and percentages for categorical variables; mean SD, and range for normally distributed quantitative variables; and median and IQR for nonnormally distributed variables. Normality will be assessed using the Kolmogorov-Smirnov test.

Comparisons between the groups of both effectiveness and safety variables will be conducted using the chi-square test for categorical variables and the 2-tailed *t* test for numerical variables. For intragroup comparisons, the McNemar test will be used for qualitative variables and the *t* test for samples related to quantitative variables. For nondichotomous variables, we will use ANOVA for parametric distributions and the Kruskal-Wallis test for nonparametric distributions. If data from quantitative variables do not follow a normal distribution, the equivalent nonparametric Mann-Whitney *U* test will be used for independent groups and the Wilcoxon signed rank test for related samples.

In all hypothesis contrasts, differences will be considered statistically significant when *P*<.05.

We will perform a single interim analysis after 50% of the sample size is enrolled. The analysis will be performed by an independent statistician, using O’Brien-Fleming limits to control for overall type I error. The trial may be stopped in case of serious adverse events related to the technique. All intermediate analyses will be conducted blinded to the principal investigators.

### Ethical Considerations

This study received clinical trials authorization from the MSCs-ES on May 27, 2024 (Clinical Trials Information System: 2024-511528-15-00), and ethics approval from the Ethics Committee for Drug Research in Galicia.

Patients scheduled for elective surgery will be provided with complete study information and will be required to sign informed consent forms during the preanesthetic consultation. Patients who are to undergo urgent surgery and therefore will not have a formal preoperative consultation will sign the informed consent form in the preoperative area.

Consent is obtained after sufficient time is given to patients to read and understand the information and to ask any questions. The informed consent form includes the option to withdraw from the study at any time. Patients who agree to participate will sign the informed consent form, which will be included in their medical record to inform postanesthesia care unit staff that the patient has been invited to participate and is enrolled in this study.

At any time during the study, patients may revoke their consent using a dedicated revocation document.

## Results

This clinical trial was approved by the European Medicines Agency on May 27, 2024, following approval by the local ethics committee earlier that month. Patient recruitment took place between June 2024 and May 2025. Statistical analysis is currently ongoing, and final results are expected to be submitted for publication in early 2026.

## Discussion

### Summary

L-ESPB has emerged as a novel and technically accessible option for postoperative analgesia in hip surgery. Although its anatomical mechanism of action suggests the potential to provide effective somatic and visceral analgesia through spread to the lumbar plexus and adjacent structures, evidence supporting its use is currently limited to case reports and small observational studies. To date, no large-scale randomized controlled trials have definitively demonstrated its efficacy in this surgical context.

This randomized controlled trial aims to fill this gap by systematically evaluating the efficacy of L-ESPB in a well-defined patient population undergoing hip surgery. By comparing postoperative opioid requirements and pain scores between patients receiving L-ESPB and those receiving standard care, this trial seeks to generate high-quality data that may inform clinical practice and contribute to the development of standardized analgesic protocols for hip surgery.

If L-ESPB is shown to significantly reduce opioid consumption and improve pain control, it could represent a valuable addition to multimodal analgesia strategies, particularly for older adults or patients who are frail and thus more vulnerable to opioid-related side effects. Conversely, if no significant benefit is observed, the results will also be important to prevent unnecessary use of a block that may add procedural time and resources without clinical gain. This trial will thus contribute meaningfully to the evidence base on regional anesthesia in orthopedic surgery and help clarify the role of L-ESPB in modern perioperative care.

Hip surgeries have major health, social, and economic implications. Regardless of surgery being prescribed to treat a fracture or coxarthrosis, patients are usually older adults with multiple associated comorbidities [1]. In this patient population, there is a tendency to undertreat pain for fear of the side effects and pharmacological interactions of conventional analgesic drugs [14]. Poor analgesic control of pain in these patients results in a slower recovery with longer hospital stays, increased incidence of chronic pain, and increased cardiovascular risks, underscoring the importance of effective analgesic strategies [15]. In this context, regional anesthesia techniques offer a promising approach to optimize analgesia while minimizing opioid consumption [16,17].

Several peripheral nerve blocks have been validated in hip surgery, such as the fascia iliaca block, femoral nerve block, lateral femoral cutaneous nerve block, PENG block, and the quadratus lumborum block, among others [18]. The fascia iliaca block has been endorsed by multiple work groups, including Klukowski et al [19], who reported that patients receiving this block required fewer analgesic interventions (3 vs 11; *P*<.01) and showed a significantly lower need for analgesics than patients without block, with no associated complications. The blockade of the iliac fascia compartment has been found to be effective in hip surgery, but it has limitations because on many occasions it fails to provide adequate analgesia of the obturator nerve, which contributes to the innervation of the most anterior capsule of the hip [20].

The PENG block, introduced in 2018 by Girón-Arango et al [21], is currently one of the most widely used peripheral nerve blocks in patients with hip fracture. Lin et al [7], who conducted a single-center, double-blind randomized comparative trial, revealed that patients who received a PENG block for intraoperative and postoperative analgesia during hip fracture surgery experienced less postoperative pain in the recovery room, but no differences were observed on the first postoperative day. Locoregional analgesia of this type is useful not only for postoperative pain control but has also been successfully applied preoperatively. Uysal et al [22] confirmed the effectiveness of preoperative femoral nerve block in trochanteric femur fracture surgery and in preventing pain during the administration of regional anesthesia.

Furthermore, given the many therapeutic analgesic options available for hip surgery, it is possible to compare them to identify the most effective approach for each type of patient and surgical technique. There is an extensive comparative bibliography on this subject, such as the randomized clinical trial conducted by Mosaffa et al [23], who concluded that the PENG block is a good method for hip fracture analgesia and provides better analgesia than the fascia iliaca compartment block.

Another block with only limited application in hip surgery, although it is more commonly used in abdominal procedures, is the quadratus lumborum block. Parras and Blanco [24], who conducted a randomized clinical trial with 104 patients with hip fracture who underwent hip hemiarthroplasty in parallel groups, reported that this block provided satisfactory analgesia for this type of intervention. However, a recent systematic review and meta-analysis found inconsistent results for its use in hip surgery, largely attributed to the unclear mechanism of action and the different approach used for each patient, with overall nonsignificant outcomes [25].

L-ESPB involves identifying the transverse apophyses at the L3-L4 level and depositing local anesthetic at this level, advancing through the erector spinae muscles to reach the apophyses. Diffusion of the local anesthetic varies depending on whether the block is performed at the thoracic or lumbar level due to the differences in the anatomy of each area. It has been found that at least 5 mL of local anesthetic would be required at the lumbar level compared to 2.5 mL at the thoracic level [26]. Some studies support the notion that L-ESPB acts similarly to paravertebral blocks, as studies performed in cadavers have shown expansion of the local anesthetic at this level. The number of intercostal spaces it may cover ranges between 3 and 7 [27].

The classic approach is the lumbar parasagittal approach at the L4 level in the ultrasound-guided plane, and it may also be performed using anatomical landmarks [28]. Tulgar and Senturk [10] and Tulgar et al [11,29], among others, reported extensive and lasting pain relief after hip arthroscopy using a single-injection L-ESPB at the L4 transverse process with 0.25% bupivacaine without significant motor block. However, these publications are limited to case series rather than randomized clinical trials. This block has even been used by Ahiskalioglu et al [30] as an intraoperative anesthetic technique together with sedation in an observational study with 15 patients considered high risk. The authors performed L-ESPB with 40 mL of a local anesthetic mixture (20 mL of 0.5% bupivacaine, 10 mL of 2% lidocaine, and 10 mL of normal saline solution) together with propofol as a sedative. All surgeries were completed satisfactorily, without conversion to general or intradural anesthesia.

Combining this block with others is feasible and has proven effective, as described by Ince et al [31], who combined the PENG block and L-ESPB, providing postoperative analgesia for a boy aged 4 years who underwent surgery for congenital hip dysplasia, reducing the need for additional analgesics.

Application of this block also allows comparison with other techniques; for example, Tulgar et al [32] conducted a prospective study and concluded that both L-ESPB and the quadratus lumborum block provided similar effects, improving analgesia quality in patients who underwent hip and proximal femur surgery compared to those who received standard intravenous analgesia.

Although several peripheral nerve blocks have been proposed in recent years for pain management in hip surgery, L-ESPB remains a little-studied technique. Most existing literature consists of case series or small observational studies, and to date, no randomized controlled trials have specifically evaluated its efficacy in this setting. We believe that this study can provide relevant and necessary information, as it offers data collected using a robust methodological design, a relatively large sample size, and blinded outcome assessment. If L-ESPB proves to be effective and safe, it could represent a practical and accessible alternative within multimodal analgesia strategies, especially for older adults or patients who are frail in whom minimizing opioid use is a key concern.

This clinical trial has major limitations, including single-center, nonblinded randomized controlled design as well as the novel yet underresearched nature of L-ESPB. Furthermore, we do not evaluate sensory blockade, which could provide valuable information for understanding its exact mechanism of action. It is also noteworthy that we include patients receiving chronic home analgesic treatment that continues to be administered during the perioperative period.

Our study has several strengths. To date, it is the only randomized clinical trial evaluating L-ESPB in patients undergoing hip surgery under either general or intradural anesthesia. With a large sample size of 180 patients compared to similar studies [18], the risk of sampling error is reduced. We adopt rigorous methods, including adjustment by regression as well as standardized intervention and follow-up protocols, which help minimize selection, performance, and observer bias.

It is likely that, upon completion of this clinical trial, further research will be necessary to build on these findings.

The results of this clinical trial will be disseminated exclusively through *JMIR Research Protocols*, and the results (including negative or inconclusive outcomes) will be reported in accordance with CONSORT (Consolidated Standards of Reporting Trials) and International Committee of Medical Journal Editors guidelines.

### Conclusions

Existing literature on the use of L-ESPB in hip surgery is scarce and inconsistent, largely relying on case reports and lacking well-designed clinical trials. If L-ESPB is shown to significantly reduce opioid consumption and improve pain control, it could represent a valuable addition to multimodal analgesia strategies.

## Abbreviations

ASA: American Society of Anesthesiologists
CONSORT: Consolidated Standards of Reporting Trials
L-ESPB: lumbar erector spinae plane block
PENG: pericapsular nerve group
SPIRIT: Standard Protocol Items: Recommendations for Interventional Trials
VAS: visual analog scale

## Data Availability

The datasets generated and analyzed during this study will be available from the corresponding author on reasonable request.
